# Supplementary material for: GLP-1 activates KATP channels in coronary pericytes as the effector of brain-gut-heart signalling mediating cardioprotection
Source: Nat Commun. 2026 Feb 14;17:2773. doi: 10.1038/s41467-026-69555-1 (PMC13018479; doi:10.1038/s41467-026-69555-1)
Supplement: Supplementary file 1 — Reporting summary [file 41467_2026_69555_MOESM1_ESM.pdf]

nature portfolio

Corresponding author(s): Svetlana Mastitskaya

Last updated by author(s): Feb 2, 2026

Reporting Summary

Nature Portfolio wishes to improve the reproducibility of the work that we publish. This form provides structure for consistency and transparency in reporting. For further information on Nature Portfolio policies, see our Editorial Policies and the Editorial Policy Checklist.

Statistics

For all statistical analyses, confirm that the following items are present in the figure legend, table legend, main text, or Methods section.

n/a	Confirmed	
☐	☒	The exact sample size (*n*) for each experimental group/condition, given as a discrete number and unit of measurement
☐	☒	A statement on whether measurements were taken from distinct samples or whether the same sample was measured repeatedly
☐	☒	The statistical test(s) used AND whether they are one- or two-sided<br>*Only common tests should be described solely by name; describe more complex techniques in the Methods section.*
☒	☐	A description of all covariates tested
☐	☒	A description of any assumptions or corrections, such as tests of normality and adjustment for multiple comparisons
☐	☒	A full description of the statistical parameters including central tendency (e.g. means) or other basic estimates (e.g. regression coefficient) AND variation (e.g. standard deviation) or associated estimates of uncertainty (e.g. confidence intervals)
☐	☒	For null hypothesis testing, the test statistic (e.g. *F*, *t*, *r*) with confidence intervals, effect sizes, degrees of freedom and *P* value noted<br>*Give P values as exact values whenever suitable.*
☒	☐	For Bayesian analysis, information on the choice of priors and Markov chain Monte Carlo settings
☒	☐	For hierarchical and complex designs, identification of the appropriate level for tests and full reporting of outcomes
☒	☐	Estimates of effect sizes (e.g. Cohen's *d*, Pearson's *r*), indicating how they were calculated

Our web collection on statistics for biologists contains articles on many of the points above.

Software and code

Policy information about availability of computer code

Data collection	Images were acquired using Zeis software (Zen 2.3). Image analysis was performed using FIJI software (ImageJ 1.53c, NIH).
Data analysis	Statistical analysis was performed using GraphPad Prism 9 software.

For manuscripts utilizing custom algorithms or software that are central to the research but not yet described in published literature, software must be made available to editors and reviewers. We strongly encourage code deposition in a community repository (e.g. GitHub). See the Nature Portfolio guidelines for submitting code & software for further information.

Data

Policy information about availability of data

All manuscripts must include a data availability statement. This statement should provide the following information, where applicable:

- Accession codes, unique identifiers, or web links for publicly available datasets
- A description of any restrictions on data availability
- For clinical datasets or third party data, please ensure that the statement adheres to our policy

Authors declare that all relevant data supporting the findings of this study are included in the paper and its supplementary information file.

Research involving human participants, their data, or biological material

Policy information about studies with human participants or human data. See also policy information about sex, gender (identity/presentation), and sexual orientation and race, ethnicity and racism.

Reporting on sex and gender	Primary cell culture was used to visualise the GLP-1 receptor expression on the cell membrane of cardiac pericytes. Sex-stratified analysis was not possible due to the limited sample size (n=2, one male and one female).
Reporting on race, ethnicity, or other socially relevant groupings	Information on race, ethnicity and other social groupings was not collected for this study.
Population characteristics	Right ventricular tissue was obtained from two pediatric patients (ages 12 and 14 years) undergoing pulmonary valve repair surgery.
Recruitment	Discarded right ventricular tissue from pulmonary valve repair surgery was obtained with informed consent from patients' legal guardians.
Ethics oversight	North Somerset and South Bristol Research Ethics Committee.

Note that full information on the approval of the study protocol must also be provided in the manuscript.

Field-specific reporting

Please select the one below that is the best fit for your research. If you are not sure, read the appropriate sections before making your selection.

☒ Life sciences ☐ Behavioural & social sciences ☐ Ecological, evolutionary & environmental sciences

For a reference copy of the document with all sections, see nature.com/documents/nr-reporting-summary-flat.pdf

Life sciences study design

All studies must disclose on these points even when the disclosure is negative.

Sample size	If a treatment causes a 40% change with a measurement standard deviation (SD) of 12% (e.g. as in Fig 2c), a power calculation (https://eda.nc3rs.org.uk/eda/powerCalculator/minimal#) indicates that, with the significance level set at 0.05, then to detect this difference with a power of 0.8, there should be at least three animals in each group. Similarly, for a response difference of 18% as in Fig 3c, with a SD of 6.6%, the number of animals needed is 4. On average we used 6 animals in each experimental group for experiments. The statistical unit for calculating s.e.m. was animals.
Data exclusions	Data were excluded from the analysis in the following cases:<br>Figure 2: if the perfusion of the heart with FITC-albumin failed completely (making the capillary perfusion analysis impossible)<br>Figure 3c-f and 4a-c: live tissue imaging: if after cannulation of the coronary artery a flow of the luminal solution was not established - in this cases, the capillaries were not adequately perfused from the very beginning of the experiment, and the experiment was discontinued in the early stages.<br>Figure 5: heart ischaemia/reperfusion experiment in vivo in mice: if the animal didn't survive till the end of the reperfusion period.
Replication	The in vivo model of heart ischaemia/reperfusion in rats and mice is highly reproducible in our hands and has been used in our previous publications (e.g. O'Farrell et al., https://pubmed.ncbi.nlm.nih.gov/29120327/). Live tissue imaging experiments were performed over a period of two years, also with consistent results: OGD consistently caused around a 20% decrease in capillary diameter at pericyte somata in cardiac tissue. This was replicated at both the UCL and the University of Bristol laboratories.
Randomization	Randomization is not relevant to the present study, since experimental animals were obtained from genetically homogeneous colonies and then assigned to different experimental groups according to the treatment/genetic status. Hearts were randomly allocated to drug and control treatments.
Blinding	All image analysis and capillary diameter measurements were performed by a person blinded to the experimental condition.

Reporting for specific materials, systems and methods

We require information from authors about some types of materials, experimental systems and methods used in many studies. Here, indicate whether each material, system or method listed is relevant to your study. If you are not sure if a list item applies to your research, read the appropriate section before selecting a response.

Materials & experimental systems

n/a	Involved in the study
☐	☒ Antibodies
☒	☐ Eukaryotic cell lines
☒	☐ Palaeontology and archaeology
☐	☒ Animals and other organisms
☒	☐ Clinical data
☒	☐ Dual use research of concern
☒	☐ Plants

Methods

n/a	Involved in the study
☒	☐ ChIP-seq
☒	☐ Flow cytometry
☒	☐ MRI-based neuroimaging

Antibodies

Antibodies used

anti-NG2 (Merck Millipore, AB5320, 1:200)
anti-NG2 (Abcam, ab275024, 1:200)
isolectin B4-Alexa Fluor 647 (Molecular Probes, I32450, 1:200)
anti-PDGFRß (R&D Systems, AF1042, 1:50)
isolectin GS-IB4-biotinylated (Life Technologies, 121414, 1:200)
anti-Kir6.1 (Alomone Labs, APC-105, 1:100)
anti-GLP-1R (Abcam, ab218532, 1:200)
anti-human PDGFRß (Santa Cruz, sc-374573, 1:50)

Validation

All immunostaining was performed according to the manufacturer's recommendations. The antibodies were tested on a positive control (e.g., pancreas tissue for GLP-1Rs); negative controls without the primary first antibody were also checked with each staining. For Kir6.1 staining of cardiac pericytes, tissue from NG2-cre/Kir6.1-flx/flx mice (conditional knockout of Kir6.1 in pericytes) was used as an additional negative control.

Animals and other research organisms

Policy information about studies involving animals; ARRIVE guidelines recommended for reporting animal research, and Sex and Gender in Research

Laboratory animals

Adult male Sprague-Dawley rats (220-250 g, or 2-3 months old) were used in this study.
Adult mice (2-3 months old) of both sexes were used for live tissue imaging and for in vivo heart ischaemia/reperfusion experiments (strains/lines used were: NG2-dsRed and NG2-CreERT2/Kir6.1flx).

Wild animals

The study did not involve wild animals.

Reporting on sex

All experiments were performed in rodents (mice and rats). For the in vivo cardiac ischaemia/reperfusion model and assessment of no-reflow following remote ischaemic preconditioning (Figure 2), only male rats were used to ensure consistency with previously published work in this well-established model (PMID:29120327; PMID:27702763), thereby enabling direct comparison and reproducibility. For subsequent in vivo mouse studies evaluating infarct size in pericyte-specific Kir6.1 conditional knockout animals (Figure 3g), an equal mix of male and female mice was used; however, sex-based analyses were not performed due to the limited availability of transgenic animals, which precluded adequately powered stratification. For live tissue imaging experiments (Figures 3b-f and 4a-c) and immunostaining (Figures 1 and 4d-g), animals of both sexes were included, but sex-specific analyses were not considered critical, as these assays interrogate conserved cellular and molecular mechanisms rather than sex-dependent physiological endpoints.

Field-collected samples

The study did not involve samples collected from the field.

Ethics oversight

All experiments were performed in accordance with the European Commission Directive 2010/63/EU (European Convention for the Protection of Vertebrate Animals used for Experimental and Other Scientific Purposes) and the UK Home Office Scientific Procedures Act (1986) with project approval from the University College London Institutional Animal Welfare and Ethical Review Committee.

Note that full information on the approval of the study protocol must also be provided in the manuscript.

Plants

Seed stocks	N/A
Novel plant genotypes	N/A
Authentication	N/A
